# Supplementary material for: Effect of Expression of Human Glucosylceramidase 2 Isoforms on Lipid Profiles in COS-7 Cells
Source: Metabolites. 2020 Nov 27;10(12):488. doi: 10.3390/metabo10120488 (PMC7761373; doi:10.3390/metabo10120488)
Supplement: Supplementary file 1 [file metabolites-10-00488-s001.pdf]

Effect of expression of human glucosylceramidase 2 isoforms on lipid profiles in COS-7 cells

Supplementary Figures

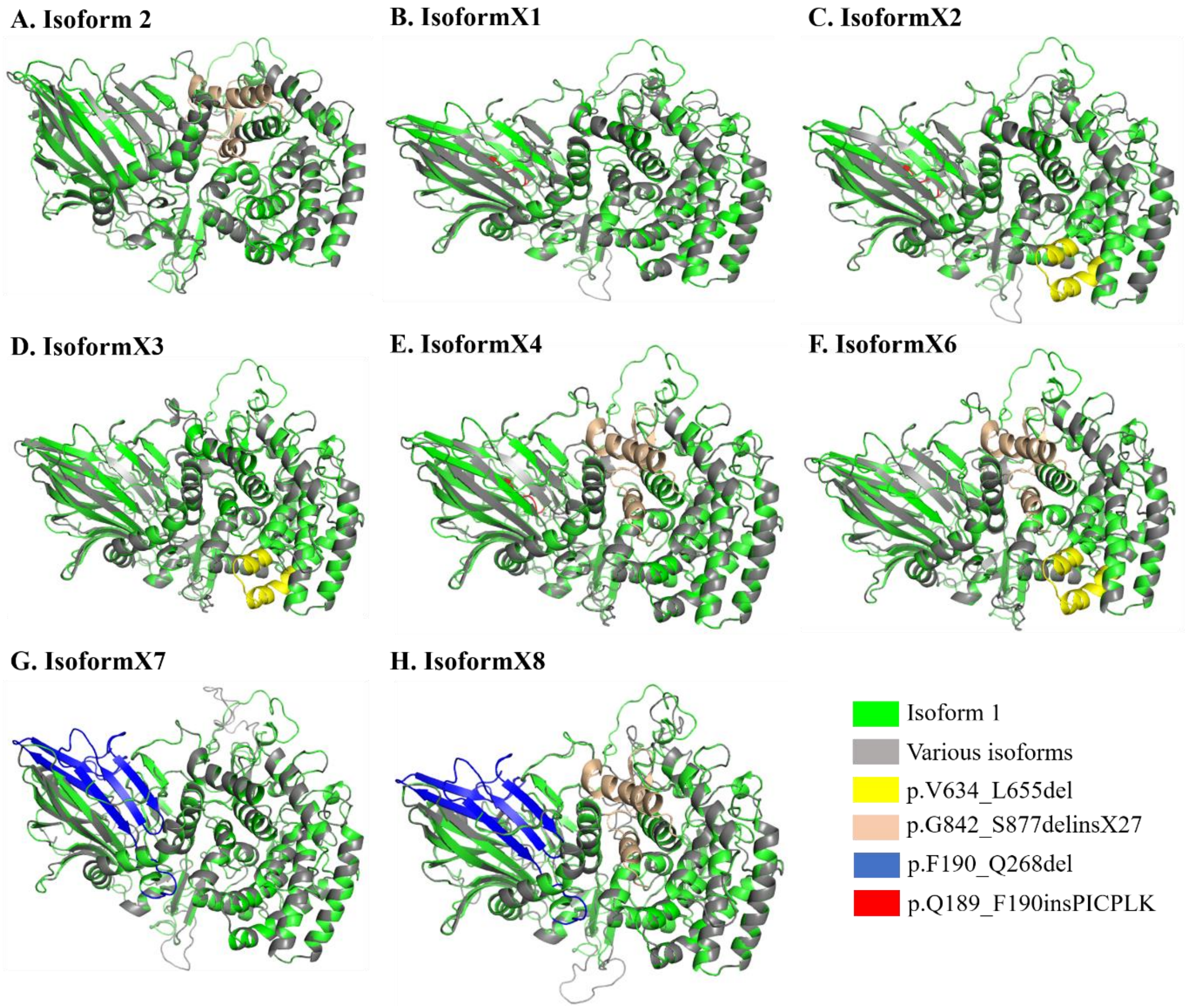

Figure S1. Superposition of homology models of various human GBA2 isoforms to GBA2 isoform 1 showing the parts missing and/or added.

In order to clarify which part are missing and/or added, the GBA2 isoform model structures were aligned to that of GBA2 isoform 1**.** The deletions are colored in the isoform 1 model, while the insertion p.Q189_F190insPICPLK is colored red in the models of isoform X1, X2 and X4.

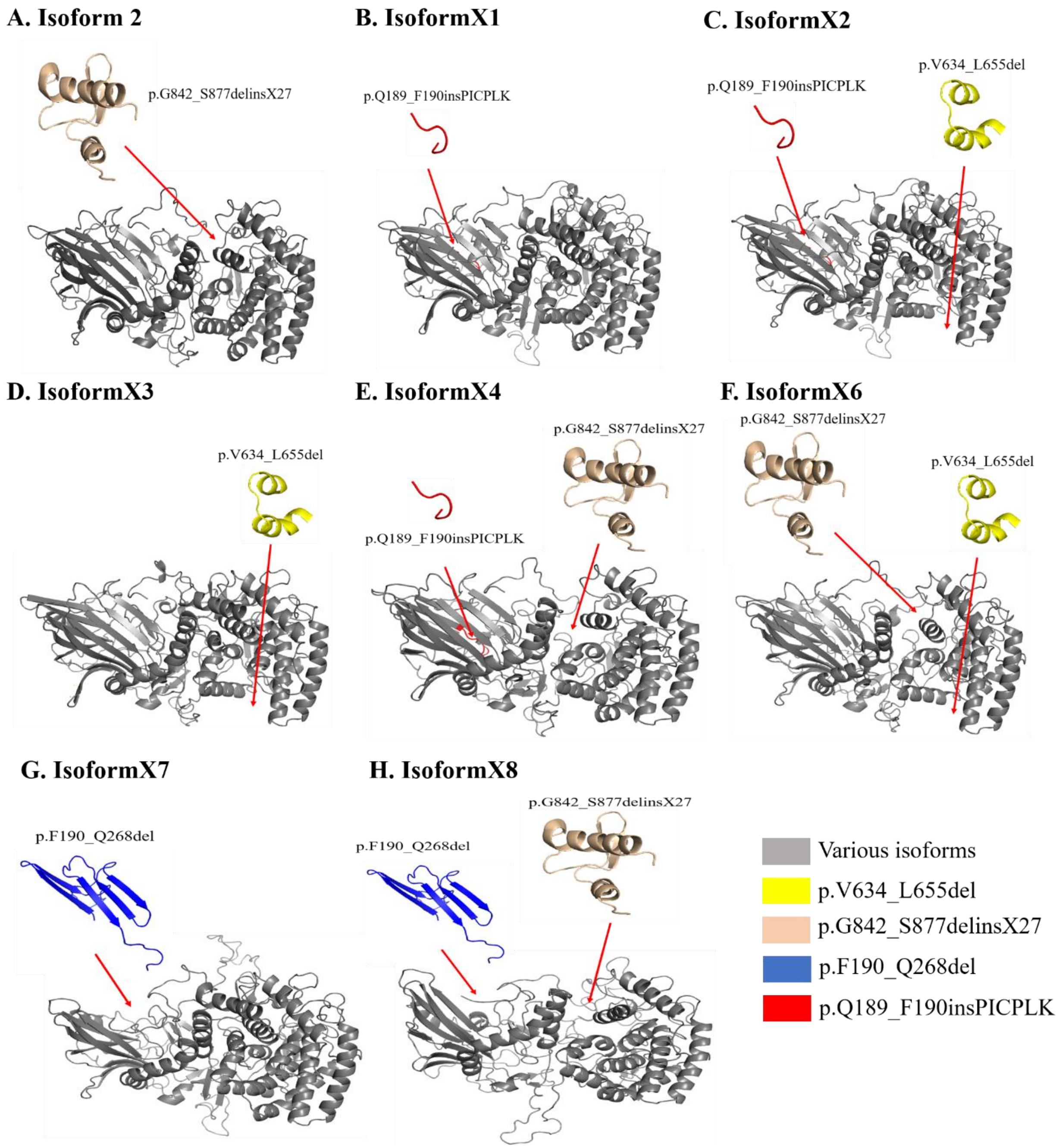

Figure S2. Homology models of human GBA2 isoforms showing the parts missing and/or added in the uncharacterized isoforms 2 to isoform X8

The model was based on the X-ray crystal structure of the *Tx*GH116 β-glucosidase (PDB code 5BVU) (30). Homology modeling of human GBA2 isoform2 to isoform X8 are shown in parts (A) to (H), respectively. The models were generated with the SWISS-MODEL server (31).

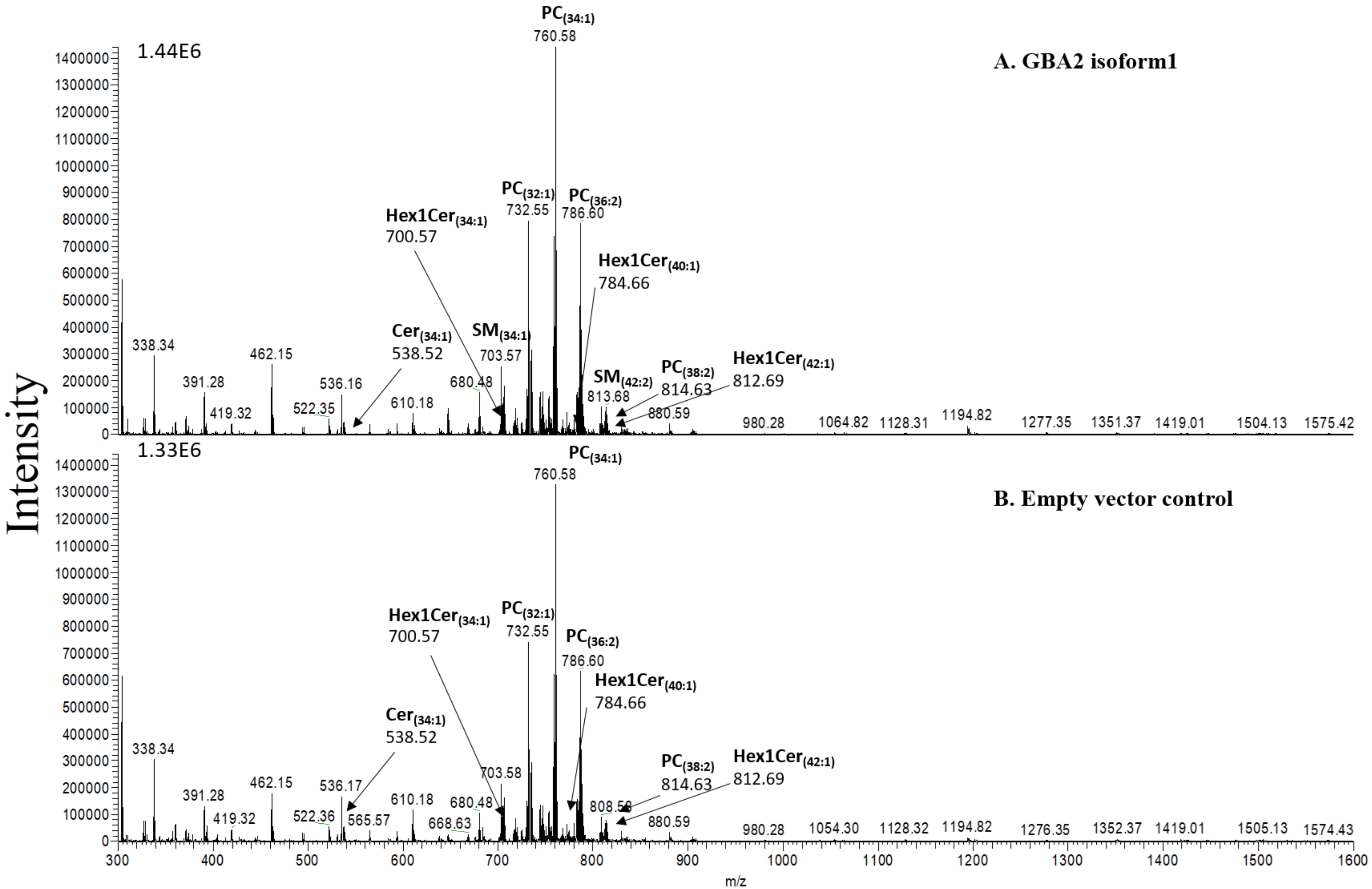

Figure S3. Positive ionization mode high resolution/accurate mass spectra with peak intensity of lipid extracts from COS-7 cells transfected with GBA2 isoform 1 (A) and empty vector control (B).

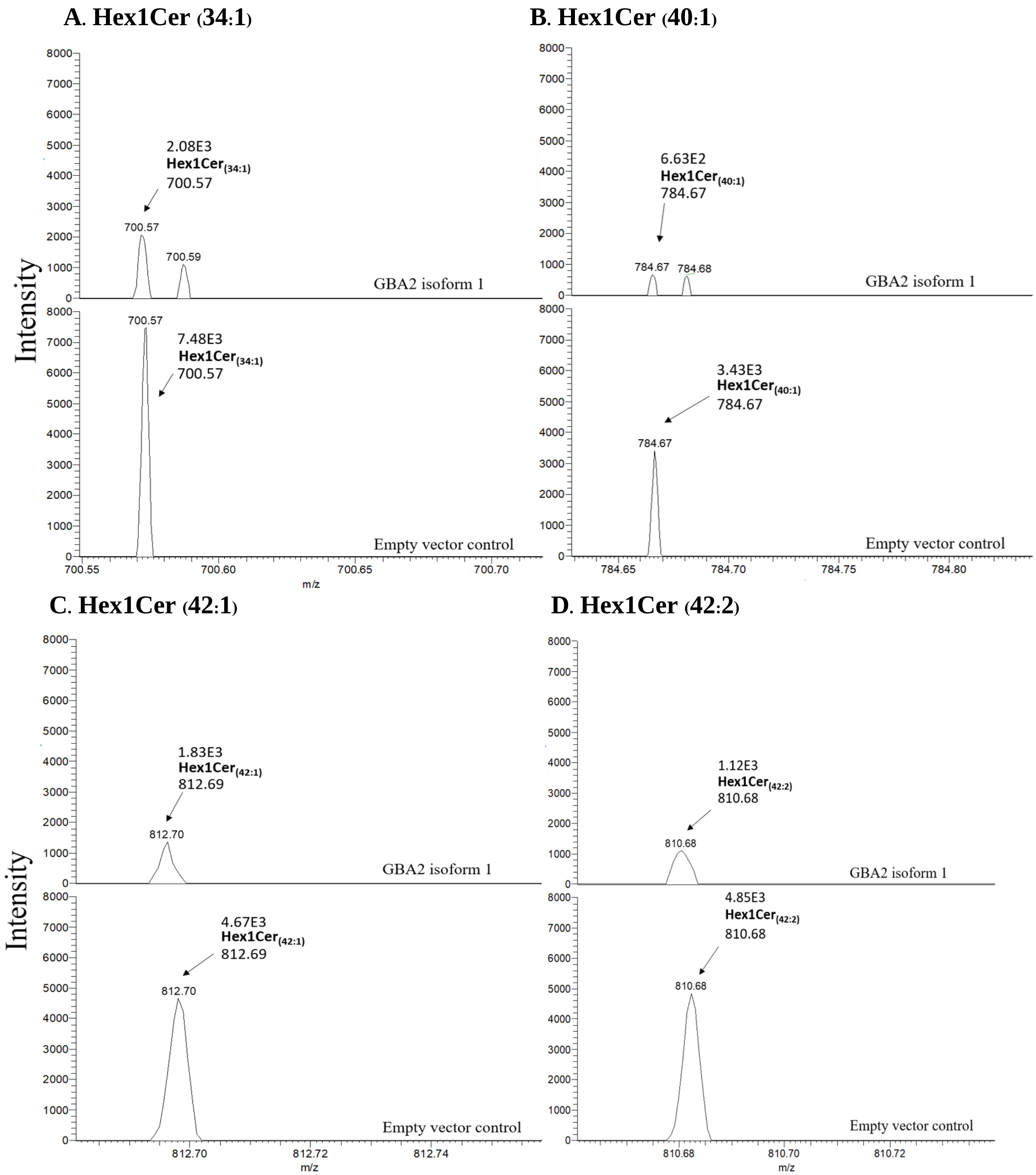

Figure S4. Mass peak intensity comparison of Hex1Cer with different lipid carbon lengths and saturation between lipid extracts of cells expressing GBA2 isoform 1 and empty vector control.

Representative mass spectra of peaks with the masses of Hex1Cer with lipid carbon compositions of 34:1, 40:1, 42:1 and 42:2 are shown in (A) to (D)

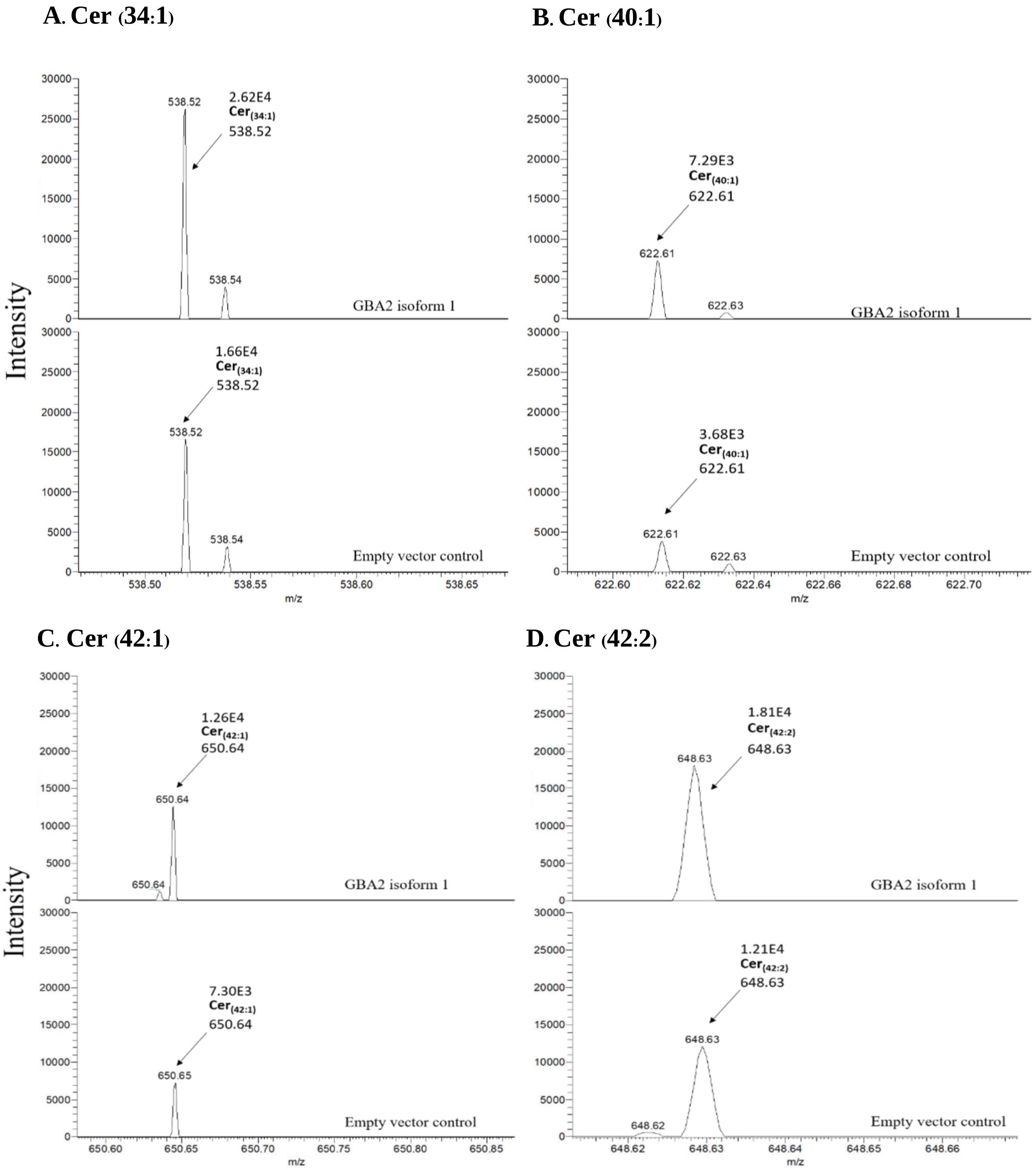

Figure S5. Mass peak intensity comparison of Cer with different carbon chain lengths and saturation between lipid extracts of cells expressing GBA2 isoform 1 and empty vector control.

Representative mass spectra of Cer with carbon compositions of 34:1, 40:1, 42:1 and 42:2 are shown in (A) to (D).

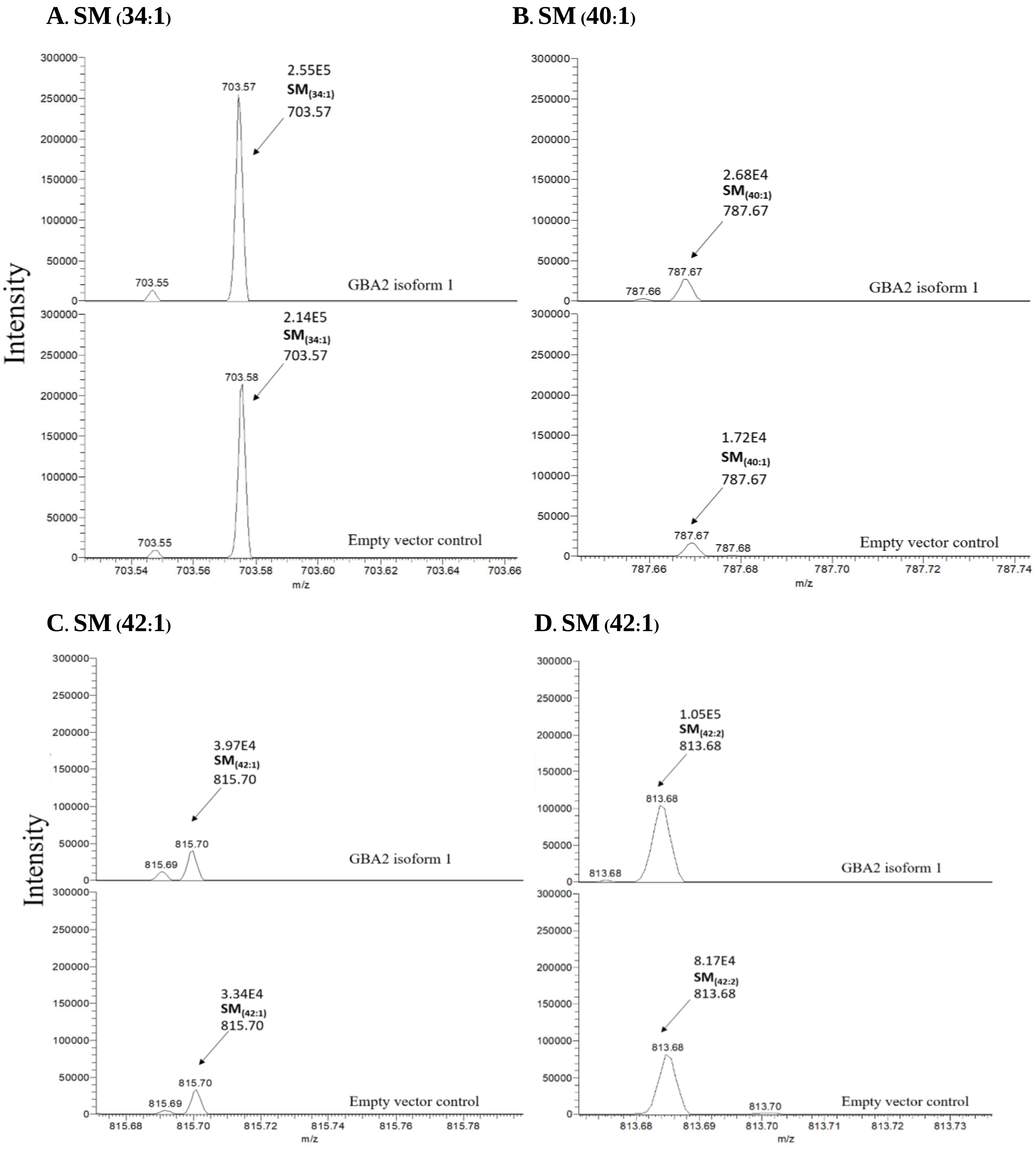

Figure S6. Mass peak intensity comparison of SM with different ceramide carbon compositions between GBA2 isoform 1 and empty vector control.

Representative mass spectra of SM with ceramide carbon compositions of 34:1, 40:1, 42:1 and 42:2 are shown in (A) to (D).

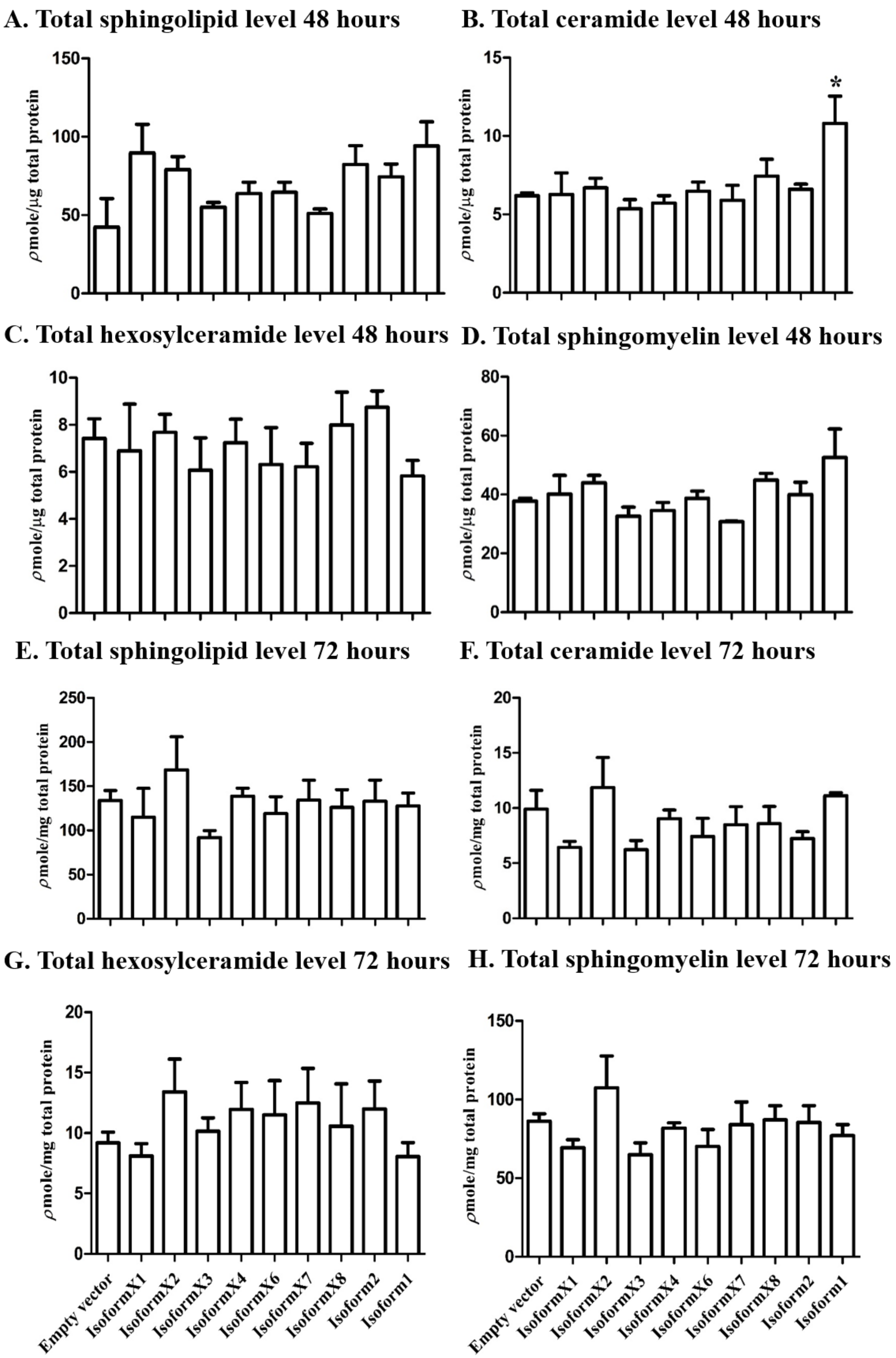

Figure S7. Profiles of total sphingolipid, total ceramides, total hexosylceramide and total sphingomyelin in COS-7 cells transfected with the 9 human GBA2 isoforms 48 (A) to (D) and 72 (E) to (H) hours post-transfection.

All species detected for each sample were included in the sum, regardless of whether they were found in other samples or not. All experiments were done with three indipendent biological replicates, and means and standard deviations are shown in A and D with * indicates differences with $p<0.05$.

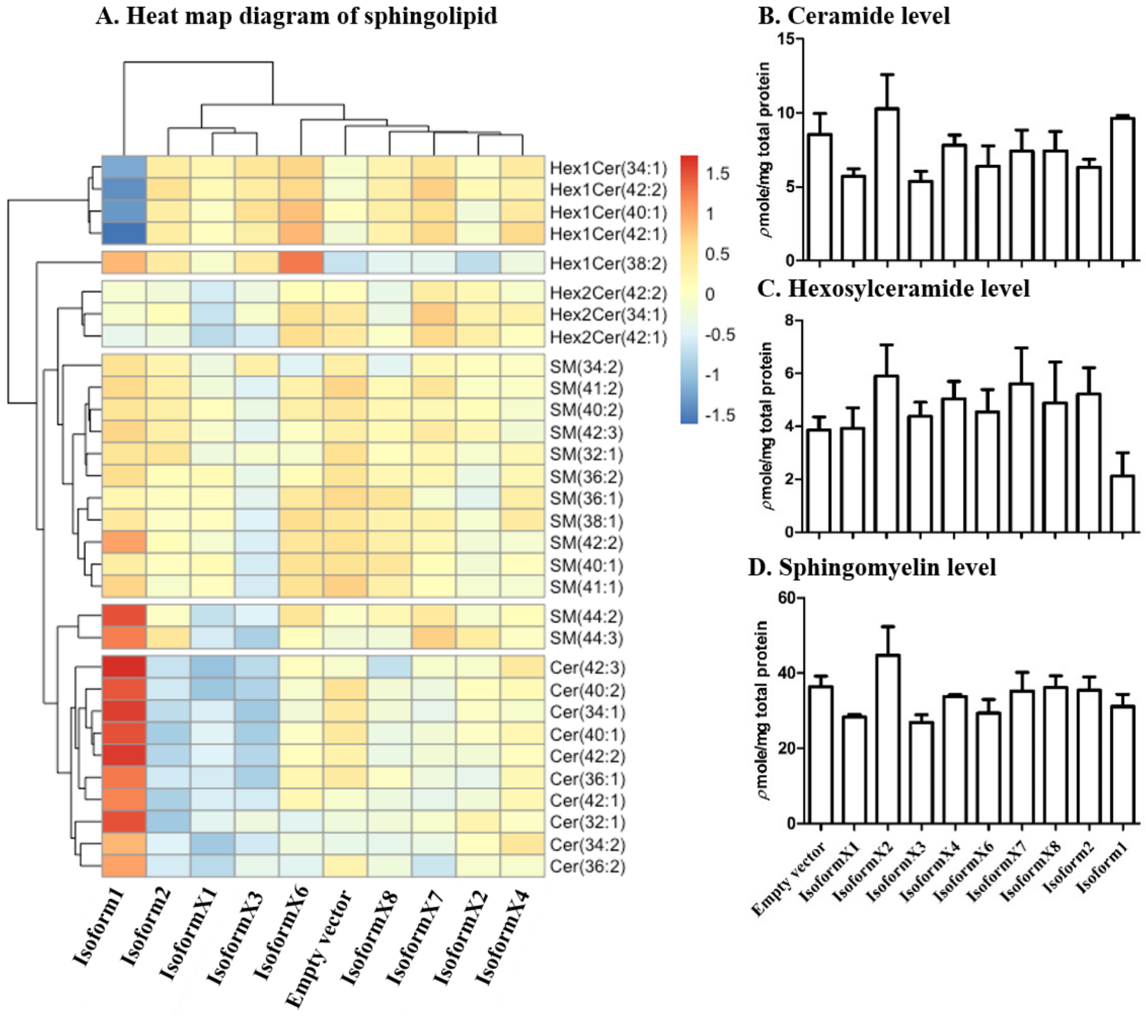

Figure S8. Relative sphingolipid levels in cells expressing respective human GBA2 isoforms at 72 h post-transfection

(A) The heat map illustrates Z-score differences from means of sphingolipid levels in cells expressing the human GBA2 isoforms and control at 72 h after transfection, while the cluster maps illustrate the similarities of the patterns. The z-scores are color-coded from blue (lower than average for that lipid species) to red (higher than average for that lipid species). **(B)** Ceramide (34:1, 36:1, 40:1, 40:2, 42:1, 42:2 and 42:3), **(C)** Hexosylceramide (34:1, 40:1, 42:1 and 42:2), and **(D)** Sphingomyelin (36:2, 40:1, 40:2, 41:1, 41:2, 42:1, 42:2, 42:3, 44:2 and 44:3), expressed as bar graphs. Values are means of three independent biological replicates. None of the differences in parts B to D reached the significance level of $p<0.05$.

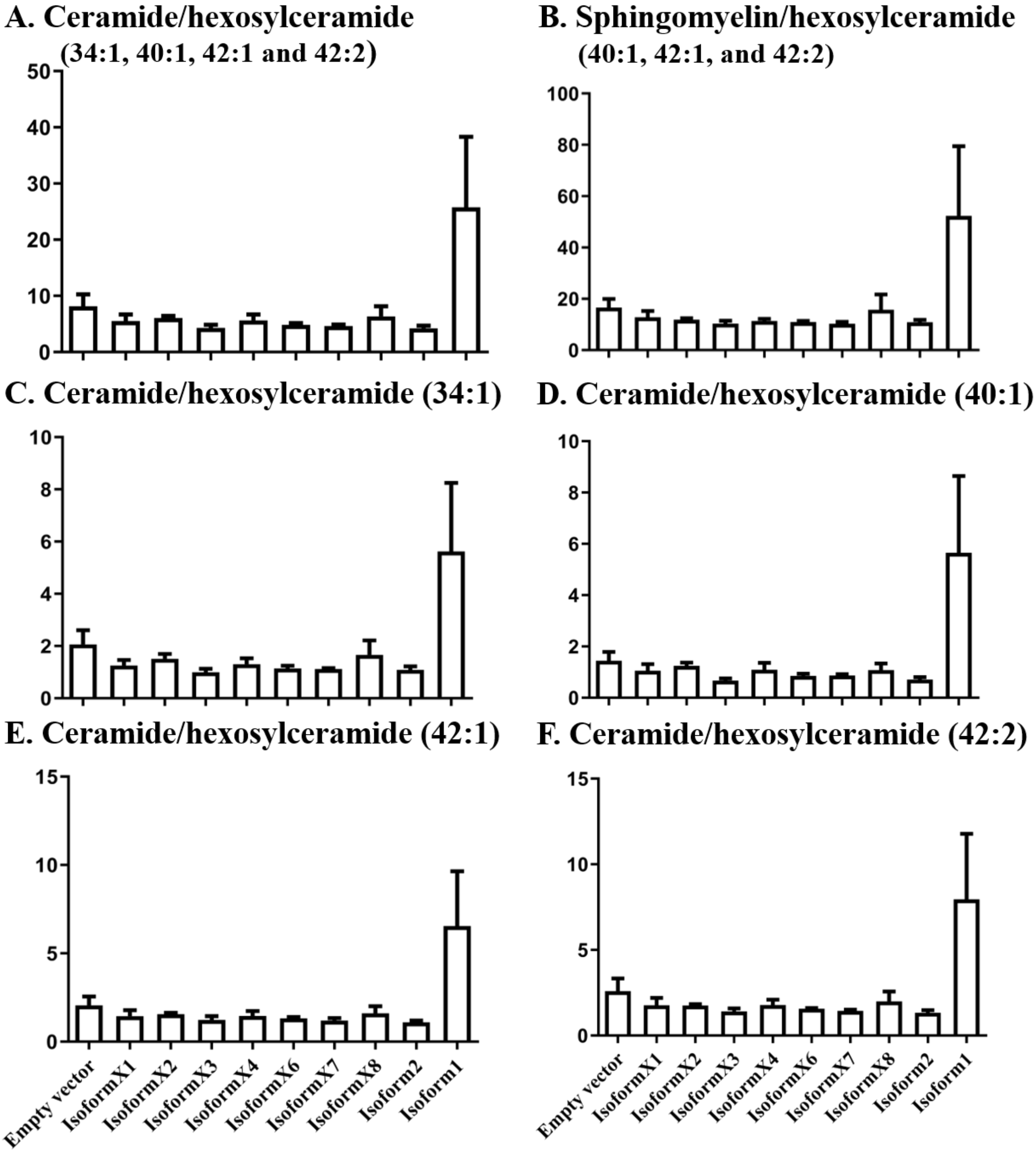

Figure S9. Effect of GBA2 isoforms on ceramide/hexosylceramide ratios of specific sphingolipid species at 72 hours post-transfection.

Sphingolipid ratio **(A)** Ceramide/ hexosylceramide (34: 1, 40: 1, 42: 1 and 42: 2), **(B)** sphingolyelin/ hexosylceramide (40:1 42:1 and 42:2) and ceramide/hexosylceramide ratios for 34:1, 40:1 42:1 and 42:2 are shown separately in **(C)**, **(D)**, **(E)** and **(F)**, respectively. Data are expressed as mean of three independent biological replicates ± SD.

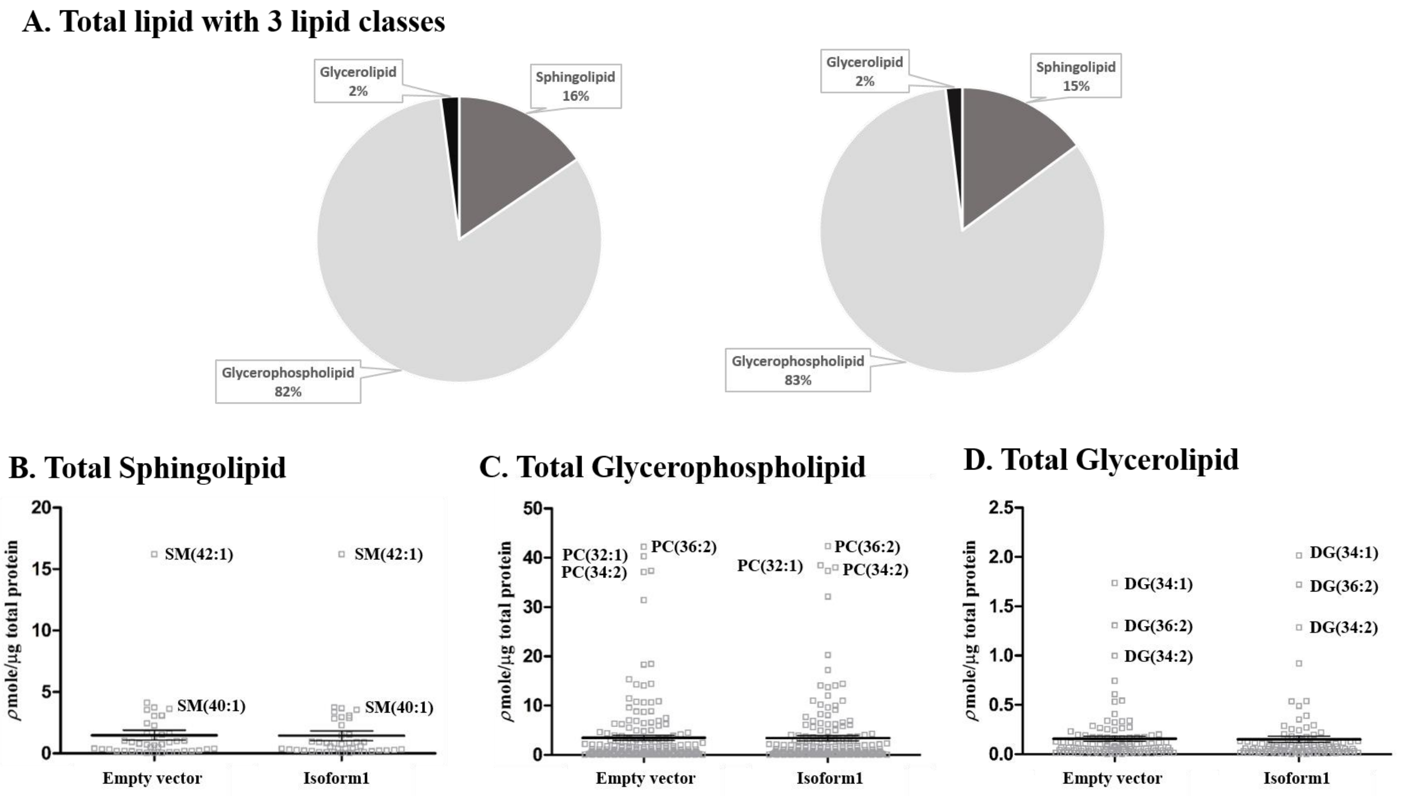

Figure S10. Relative levels of sphingolipids, glycerophospholipids and glycerolipids in COS-7 cells transfected with empty vector and vector for human GBA2 isoform 1 for 72 hours.

(A) Relative amounts of 3 classes of lipid species. Levels of specific sphingolipid **(B)** glycerophospholipid **(C)** and glycerolipid **(D)** species and average values in control and cells expressing GBA2 isoform 1 are illustrated as parallel dot plots. Amounts were determined by mass spectrometry analysis of COS- 7 cell lipid extracts. Result are representative of three independent replicates, $*p<0.05$.

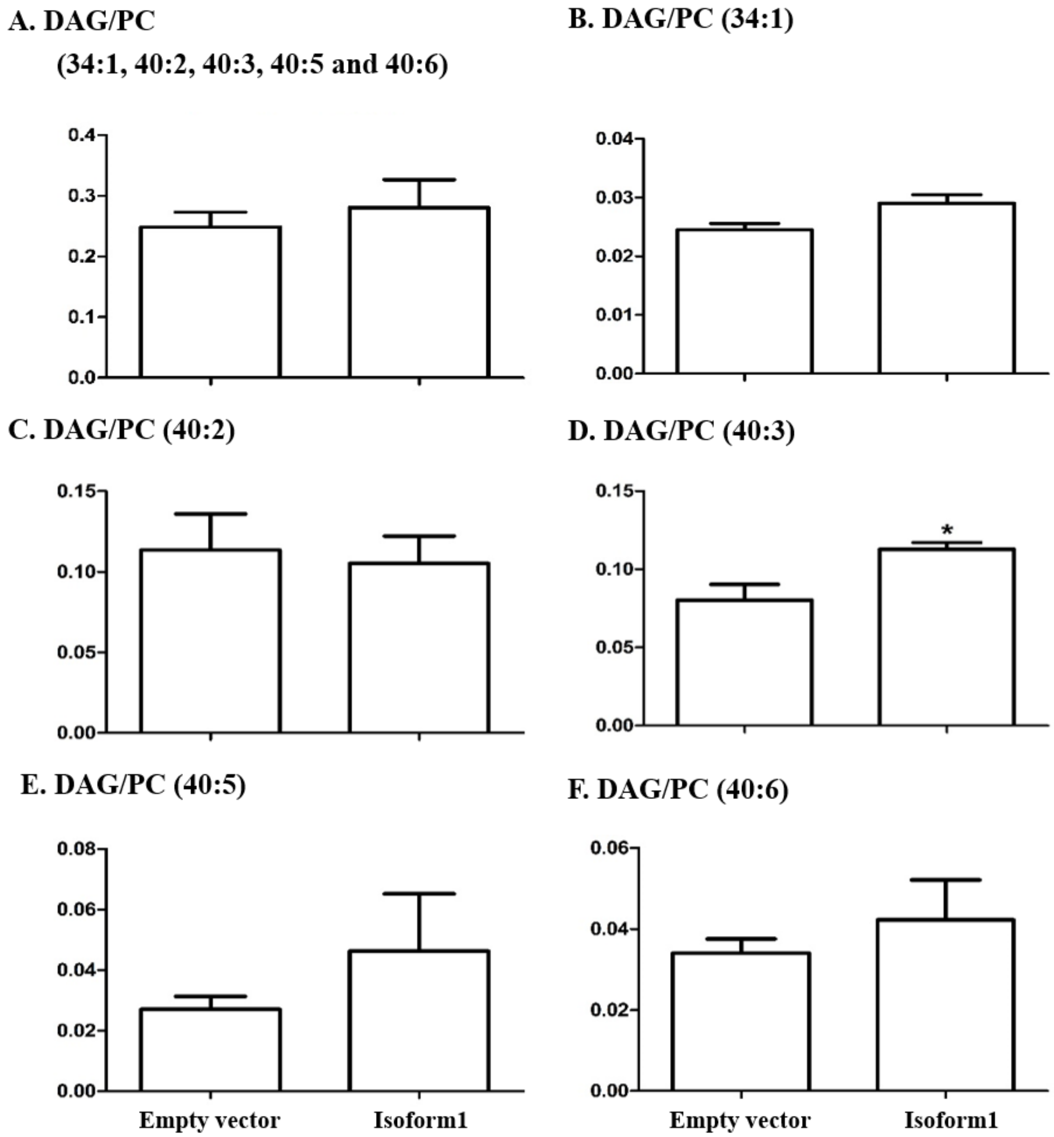

Figure S11. Ratios of diacylglycerol to phosphatidylcholine in COS-7 cells transfected with control vector and GBA2 isoform 1 expression vector after 72 hours.

The ratios of diacylglycerol (DAG) species to phosphatidylcholine (PC) for those lipid species found in both lipid classes (34:1, 40:2, 40:3, 40:5 and 40:6) are shown in **(A)**. The individual DAG/PC ratios for 34:1, 40:1, 42:1 and 42:2 are shown in **(B)**, **(C)**, **(D)**, **(E)** and **(F)**, respectively. Amounts in lipid extracts of COS-7 cells transfected with empty vector and human GBA2 isoform 1 expression vector for 72 hours were determined by shotgun mass spectroscopy analysis. Data are expressed as mean of three independent biological replicates ± SD, * indicates $p<0.05$ in the unpaired t-test. The distributions of sample values for all conditions did not deviate significantly from a normal distribution in a Shapiro-Wilk test.

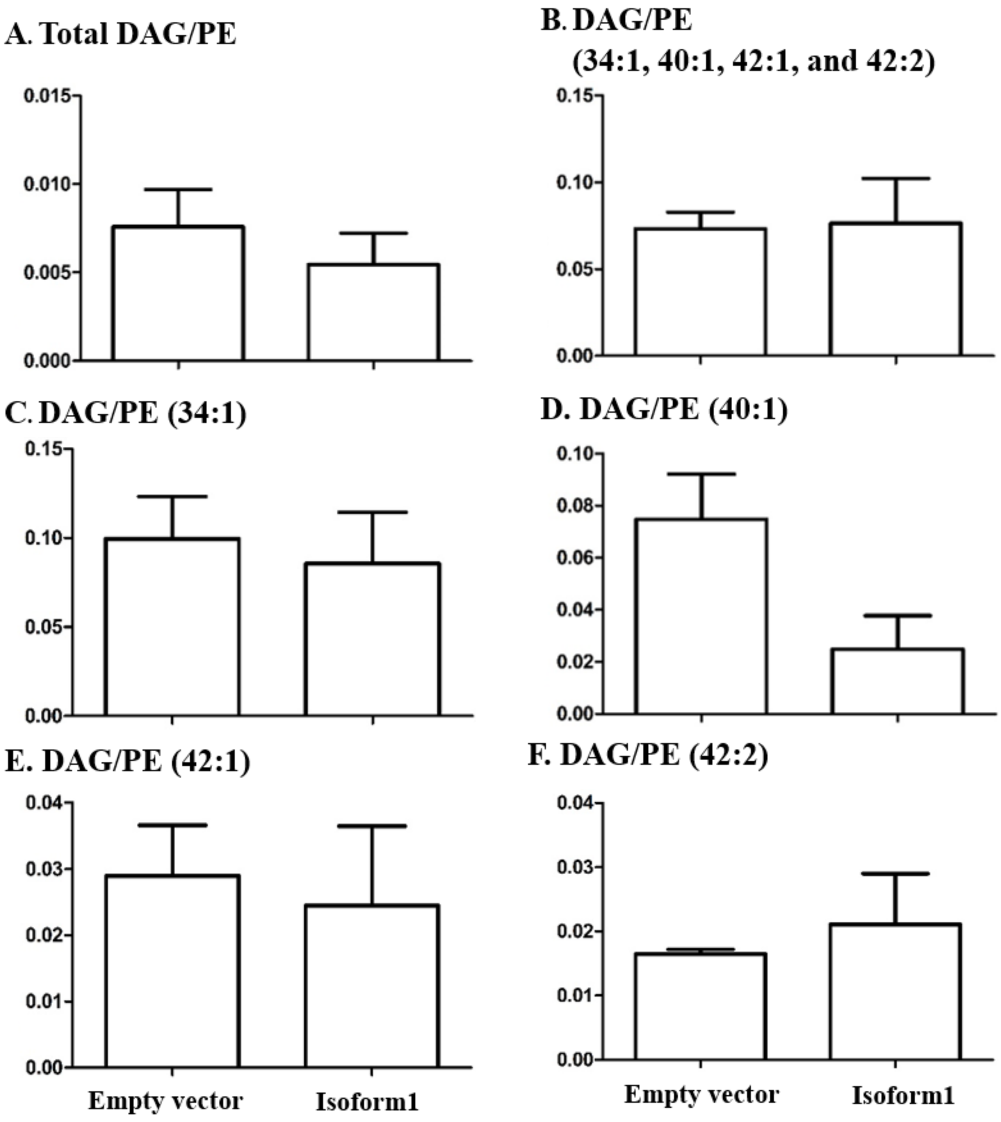

Figure S12. Relative levels of diacylglycerol to phosphatidylethanolamine in COS-7 cells transfected with control vector and GBA2 isoform 1 expression vector for 48 hours.

The ratios of total diacylglycerol (DAG) species to phosphatidylethanolamine (PE) species are shown in **(A)** DAG/PE for those lipid species found in both lipid classes (34:1, 40:1, 42:1, and 42:2) are shown in B. The individual DAG/PE ratios for 34:1, 40:1, 42:1 and 42:2 are shown in **(C)**, **(D)**, **(E)**, and **(F)**, respectively. Amounts in lipid extracts of COS-7 cells transfected with empty vector and human GBA2 isoform 1 expression vector for 48 h were determined by mass spectrometry analysis. Means and standard deviations of three independent biological replicates are represented.

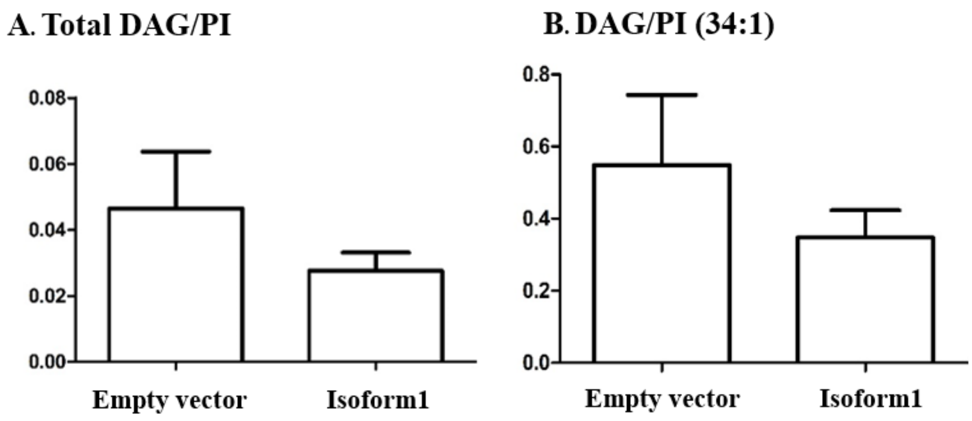

Figure S13. Ratios of relative levels of total diacylglycerol to phosphatidylinositol and phosphatidylethanolamine in COS-7 cells transfected with control vector or GBA2 isoform 1 expression vector for 48 h.

The ratios of total diacylglycerol (DAG) species to phosphatidylinositol (PI) species are shown in **(A)** DAG/PI for lipid species 34:1, the only species found in both lipid classes is shown in **(B)**. Data are expressed as mean of three independent biological replicates ± SD.

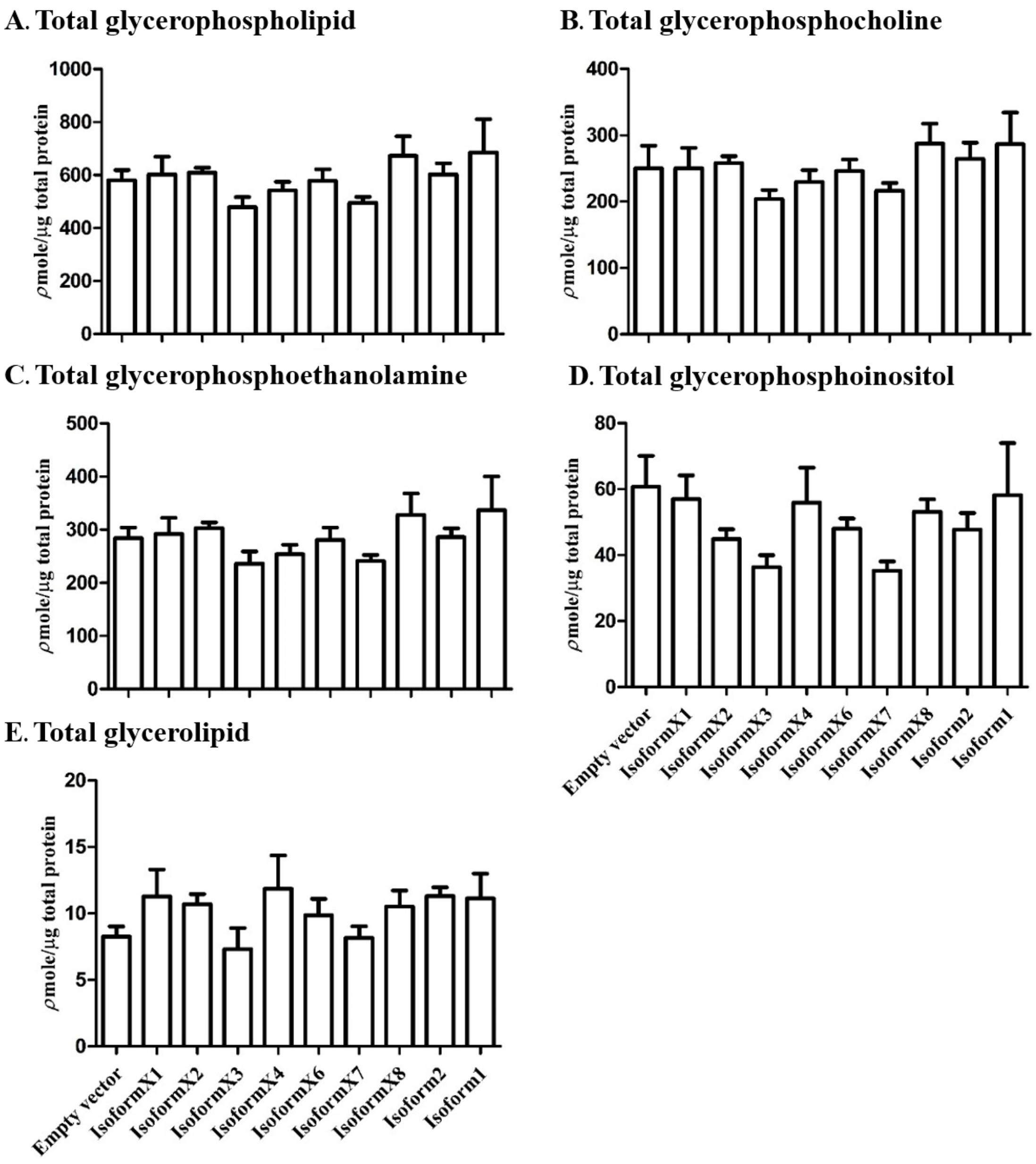

Figure S14. Comparison of sphingolipid, glycerophospholipid and glycerolipid in COS-7 control cells and cells transfected with the human GBA2 9 isoforms 48 hours.

The levels of glycerophospholipids (A), phosphatidylcholine (B), phosphatidyl ethanolamine (C), phosphatidlylinositol (D) and glycerolipids (D) after 48 hour transfection with vectors expressing the indicated GBA2 isoforms. Data are expressed as mean of three independent biological replicates ± SD**.**
